# Intermittent plasticity in individual grains: A study using high energy x-ray diffraction

**DOI:** 10.1063/1.5068756

**Published:** 2019-01-07

**Authors:** K. Chatterjee, A. J. Beaudoin, D. C. Pagan, P. A. Shade, H. T. Philipp, M. W. Tate, S. M. Gruner, P. Kenesei, J.-S. Park

**Affiliations:** 1 Mechanical Science and Engineering, University of Illinois, Urbana-Champaign, Illinois 61801, USA; 2 Cornell High Energy Synchrotron Source (CHESS), Cornell University, Ithaca, New York 14853, USA; 3 Materials and Manufacturing Directorate, Air Force Research Laboratory, Wright-Patterson Air Force Base, Ohio 45433, USA; 4 Laboratory of Atomic and Solid State Physics, Cornell University, Ithaca, New York 14853, USA; 5 Kavli Institute for Nanoscale Science, Cornell University, Ithaca, New York 14853, USA; 6 Advanced Photon Source, Argonne National Laboratory, Lemont, Illinois 60439, USA

## Abstract

Long-standing evidence suggests that plasticity in metals may proceed in an intermittent fashion. While the documentation of intermittency in plastically deforming materials has been achieved in several experimental settings, efforts to draw connections from dislocation motion and structure development to stress relaxation have been limited, especially in the bulk of deforming polycrystals. This work uses high energy x-ray diffraction measurements to build these links by characterizing plastic deformation events inside individual deforming grains in both the titanium alloy, Ti-7Al, and the magnesium alloy, AZ31. This analysis is performed by combining macroscopic stress relaxation data, complete grain stress states found using far-field high energy diffraction microscopy, and rapid x-ray diffraction spot measurements made using a Mixed-Mode Pixel Array Detector. Changes in the dislocation content within the deforming grains are monitored using the evolution of the full 3-D shapes of the diffraction spot intensity distributions in reciprocal space. The results for the Ti-7Al alloy show the presence of large stress fluctuations in contrast to AZ31, which shows a lesser degree of intermittent plastic flow.

## I. INTRODUCTION

Plastic deformation in metals occurs by intermittent motion of dislocations as has been shown in many experimental settings. Early observations of metal deformation proceeding in discrete steps are detailed in reviews by Argon^1^ and Maass and Derlet.^2^ The motion of dislocations progresses in “bursts” that vary in size (both spatial extent and the number of dislocations participating) and temporal frequency. Accompanying this dislocation motion is a release of elastic energy (relaxation) producing drops in the local stress level and the driving forces for dislocation motion which may be associated with corresponding changes (both increases and decreases) in the stress states of neighboring grains in a polycrystalline aggregate.^3^ At the atomic scale, new dynamic transmission electron microscopy measurements have been able to directly image intermittent dislocation motion,^4,5^ while techniques such as acoustic emission and compression of micropillars offer rich datasets to characterize the intermittency of strain bursts.^6–8^ Although a thorough review is outside the scope of the present paper, this area of inquiry is well-served by several sources,^1,2,9,10^ providing a detailed background from different perspectives.^11^ While a great deal of work has been done, simultaneous measurements to directly connect dislocation motion to stress relaxation have proven to be difficult, and even more so for grains embedded in the bulk of a polycrystal. The present work makes an advance in linking the stress state and intermittent slip, critical for advancing the constitutive models used for metal plasticity, through utilization of newly available high rate x-ray data.

The probing of isolated diffracted intensity (spots) from individual grains provides a natural avenue for studying stress relaxation and dislocation motion simultaneously: radial shifts of diffraction spot centroids are related to changes in the average elastic strain state in a grain,^12^ azimuthal shifts are associated with plasticity induced lattice rotations,^13^ and the spread of the distribution of intensity in 3-D has significant contributions from the dislocation content present.^14^ While natural, these measurements have not been possible because x-ray sources and detectors were not capable of probing large enough regions of reciprocal space with a sufficient time resolution. This is no longer the case due to recent advances in detector technology. In this work, a high speed mixed mode pixel array detector (MM-PAD) was utilized to capture wide dynamic range diffraction data at image (frame) rates of ∼300 Hz during stress relaxation. Such temporal resolution was necessary for following the evolution of the diffraction spots, so as to identify bursts of plasticity within individual grains. The study of transients using the MM-PAD was complemented by far-field High energy Diffraction Microscopy (ff-HEDM),^15–17^ to connect intermittent plasticity with the full stress state of individual grains within a polycrystalline aggregate. Two hexagonal metals, Ti-7Al, and the magnesium alloy, AZ31, were studied. Intermittency in both of these alloys is promoted by interactions between multiple dislocation types and the presence of alloying elements.

The organization of the paper is as follows. Procedures common to the two materials are given in Sec. II, with some limited detail specific to a particular alloy are given as part of Sec. III. Two directions are taken in the presentation of findings in Sec. III:

• First, specific examples of transient dislocation evolution are examined through changes in diffraction intensity on the MM-PAD, and related to specific grains; for these few grains, the ff-HEDM analysis is used for identification of the orientations and stress tensors, to draw association of observed transients with the tensorial state of stress driving slip;
• Then, scaling relationships are developed between the size and the probability distribution transients over the course of repeated stress relaxation steps, for diffraction spot centroid motions tracked on the MM-PAD detector.

The experimental techniques and the results provide insight into the material deformation behavior at the scale of individual grains and hold promise for improving investigation methods and modeling of crystal plasticity.

## II. MATERIALS AND METHODS

### A. Samples

Samples were prepared from Ti-7Al and AZ31. The Ti-7Al material was extruded and annealed to produce equiaxed grains of size 80–100 *μ*m with a minimal substructure.^18^ This microstructure is conducive for generating separately distinguishable, intense diffraction spots. A tensile specimen of cross-section 1 mm × 1 mm and gauge length 10 mm was prepared from this material. As compared to the Ti-7Al, the AZ31 material exhibited a finer grain size of roughly 25 *μ*m, with many more, but less intense, diffraction spots. The AZ31 sample was fabricated from a 12.7 mm thick annealed and rolled plate.^19^ The nominal cross-section of this sample was 2 mm × 2 mm and the gauge length was 10 mm.

### B. High energy x-ray diffraction experiment with MM-PAD

**FIG. 1.**
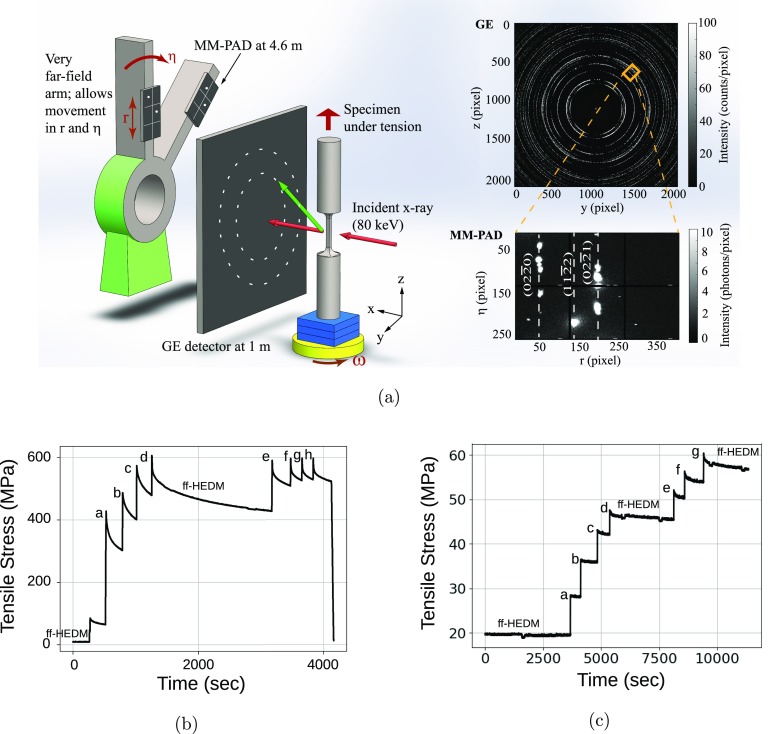
High energy x-ray diffraction experiments conducted with Ti-7Al and AZ31 samples using a combination of a single GE detector and high speed MM-PAD (2 × 3 array of chips). Given in (a) is a schematic of the experimental setup used at the Advanced Photon Source beamline 1-ID to study intermittency in plasticity. Note the very far-field location of the mixed mode pixel array detector (MM-PAD). Diffraction rings on the GE detector (2048 pixels × 2018 pixels) and a few diffraction spots on the 2 × 3 module of the MM-PAD (256 pixels × 384 pixels) generated for a Ti-7Al alloy sample are shown in the rightmost panels: the orange square (top, right) indicates the region that projects onto the MM-PAD (bottom, right). The nominal stress from the load cell measurement during loading and relaxation of (b) scans a–h of Ti-7Al and (c) scans a-g of AZ31. Times at which diffraction data for ff-HEDM are indicated in the stress relaxation plots.

High energy x-ray diffraction experiments were performed at the 1-ID-E endstation of the Advanced Photon Source [Fig. 1(a)]. The use of a monochromatic beam in this experimental setting supported a rotating crystal analysis with *in situ* loading. The key experimental advance in the present work is the tracking of diffraction transients while oscillating the sample about a rotation axis aligned with the loading direction, so as to capture the changes in reciprocal space associated with plastic slip and concomitant accumulation of lattice defects. Oscillating the polycrystalline sample under load enables study of the temporal evolution of isolated diffraction spots in 3D reciprocal space (not to be confused with the more typical line profile analysis of powder patterns from a polycrystalline aggregate). As the peaks are associated with specific grains, rapid evolution of spot position and shape can be connected to isolated plasticity events within a grain. To acquire images in the rotating crystal configuration with a high energy monochromatic beam, an MM-PAD equipped with CdTe sensors was used to acquire x-ray images at frame rates of up to 300 Hz.

**FIG. 2.**
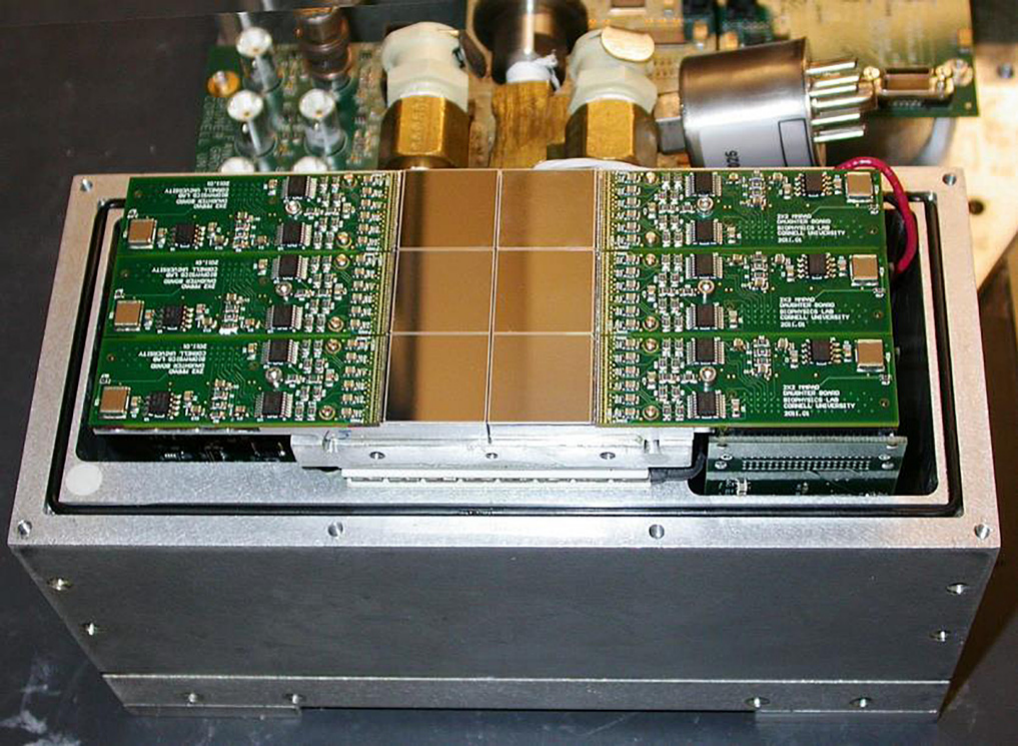
The MM-PAD is shown with the vacuum cover and x-ray window removed. The 2 × 3 arrangement of detector modules are the brownish squares in the center. Each module consists of 128 × 128 square pixels, where each pixel is 150 *μ*m of a side. Each module is roughly 2 cm × 2 cm in size. There is a 5 pixel wide (0.75 mm) inactive area between adjacent modules. (This photo is of an MM-PAD with Si, instead of CdTe sensors; otherwise, the two types of MM-PADs look identical.)

The MM-PAD^20^ is a fast-framing detector developed at Cornell University that is capable of capturing images at rates as high as 1 kHz while still maintaining excellent sensitivity over a range from single x-rays/pixel/frame to ∼8 × 10^6^ x-rays/pixel/frame. Figure 1(a) shows the mounting of the MM-PAD and the instrument geometry used for the experiments. The MM-PAD version used here was equipped with 0.75 mm thick CdTe x-ray sensors capable of efficiently detecting the 80.725 keV x-rays used in this study.^21,22^ This MM-PAD (Fig. 2) consists of a 2 × 3 arrangement of detector modules, where each module is composed of 128 × 128 pixels, and each pixel has an area of 150 *μ*m × 150 *μ*m, resulting in 256 × 384 active pixels. There is an inactive space of about 0.75 *μ*m width between adjacent detector modules. The MM-PAD was mounted on a very far-field arm at 4.6 m from the sample that was suitable for translating and rotating the detector to different radial (*r*) and azimuthal (*η*) positions so as to capture desired diffraction spots [Fig. 1(a)]. This detector was placed at *η* angles of 21.9° and 6.3° for the Ti-7Al and AZ31, respectively. During measurements, the sample was rotated by the angle *ω* about the *z* axis.

The choice of an incident x-ray of energy 80.725 keV served to “compress” reciprocal space (to fit more reflections on the MM-PAD detector area), while maintaining a detector distance affording resolution of diffraction vector changes of ∼2 × 10^−5^. Uniaxial tensile specimens were prepared from Ti-7Al and AZ31 materials. Testing was performed at room temperature using a load frame that is stiff with respect to sample compliance. Using displacement control, a load increment followed by a dwell was applied to the sample to develop a stress relaxation response, and this “displacement-hold” procedure was repeated several times. MM-PAD scans were performed during stress relaxation steps [as indicated in Figs. 1(b) and 1(c)] while concurrently oscillating the sample about ±2.5° in *ω* at a frequency of 2 Hz, with frames triggered by the rotating stage. The combination of an area detector with the sweeping stage oscillations provides a full 3-D intensity distribution of the diffraction spot. In the following, a “cycle” refers to a single oscillation in *ω*, with 146 total images captured on the MM-PAD: 73 during the forward rotation and 73 during the reverse rotation. Images were collected for 68 and 136 such cycles for the Ti-7Al and AZ31, respectively. Hence, a total of 9,928/19,856 images were collected for each Ti-7Al/AZ31 stress relaxation event, providing for tracking of diffraction spot evolution in 2*θ*, *η*, and *ω*. The beam size was set to illuminate a significant volume, while having distinct and isolated diffraction spots: 1.5 mm (H) × 1 mm (V) for Ti-7Al and 2 mm (H) × 100 *μ*m (V) for the finer-grained AZ31. For the purpose of data analysis, images from the forward and reverse directions of oscillation were summed at positions of corresponding *ω*, giving a stack of 73 images for each cycle.

A single amorphous silicon flat panel GE detector (2048 × 2048 pixels covering 41 cm × 41 cm), placed at a distance of 1m from the sample [Fig. 1(a)] was used to achieve more complete coverage of reciprocal space for ff-HEDM analysis. The GE detector was translated in front of the MM-PAD and removed when the MM-PAD was used. Images were collected for *ω* steps of 0.25° through a rotation of 360° at times indicated in Figs. 1(b) and 1(c). The macroscale, or sample, stress shown in Figs. 1(b) and 1(c) was computed from load cell data collected at 1 s intervals. For the Ti-7Al sample, the beam size for collecting data on the far-field detector was the same as that used for the MM-PAD; the vertical beam size was opened to 200 *μ*m for the AZ31 specimen. The software package hexRD^23^ was used to analyze the large panel area detector images, giving grain orientations, positions and strain information. Spots found on the MM-PAD were directly correlated with the grains identified through the ff-HEDM analysis. The diffraction spots found on the MM-PAD were matched to an orientation found using ff-HEDM by determining the crystallographic fiber associated with the spot and finding the orientation that lies on the fiber. Spread of diffraction spots as a consequence of plastic deformation, and the finer grain size of the AZ31 placed demands on associating groups of diffraction spots with specific orientations (indexing) of grains to determine orientation and strain. Lattice orientation and strain, for particular grains associated with diffraction spots on the MM-PAD images were fit with relatively close tolerances, to achieve smaller error at the expense of using fewer diffraction spots to determine strains. Linear elasticity was used to obtain the stress tensor from lattice strain, with elastic constants from Refs. 24, 19, and 25 for Ti-7Al and AZ31, respectively.

### C. Intermittency data processing

The presence and motion of dislocations in the crystal leads to a change in the diffraction spot intensities;^14^ therefore, this measure is taken as a starting point for studying plasticity bursts. To identify plastic events, the first step is to take the difference between the photon intensities for sequential MM-PAD images. The intensity fluctuation data are extracted from the stress relaxation periods only; data from the displacement increment are omitted. For each reflection, pixel locations where there is a decrease in intensity are tabulated, the notion being that such decreases would be associated with a spread in orientation following from the lattice rotation associated with dislocation motion. Clusters of pixels from the list are identified using the DBSCAN algorithm,^26^ as implemented in Scikit-learn.^27^ The distance tolerance used in DBSCAN was prescribed such that clusters are formed of adjacent pixels showing an intensity decrease, and it is therefore possible to have multiple clusters in a single diffraction spot.

It is indeed possible to proceed and develop statistics based solely on changes in intensity (an example is provided in the Appendix). However, this choice leads to issues of relating grains of different size or comparing transients from different (*hk*.*l*) reflections. As an alternative, the average diffraction vector is evaluated for each pixel cluster at time *t* as

$$\bar{g}(t)=\frac{\sum_{k=0}^{m}g(p_{k})I(p_{k},t)}{\sum_{k=0}^{m}I(p_{k},t)}$$ (1)

for the *m* pixels with coordinates *p* in the cluster, weighted by intensity *I*(*p_k_*, *t*). Two measures, based on the difference in diffraction vectors between sequential measurements, are developed for the statistical analysis. The change in diffraction vector length, associated with lattice strain, is taken as

$$\Delta g_{L}=\frac{|g(t){|}_{2}-|g(t-\Delta t){|}_{2}}{|g(t-\Delta t){|}_{2}},$$ (2)

where $|\cdot {|}_{2}$ is the vector 2-norm. This length change for a particular diffraction vector is added to a list of events, provided Δ*g_L_* > 0. That is, an increase in diffraction vector length in reciprocal space is indicative of a lattice strain relaxation event. The lattice reorientation associated with plastic deformation is characterized through the change in angle as

$$\Delta g_{A}={\text{cos}}^{-1}\left[\hat{g}(t)\cdot \hat{g}(t-\Delta t)\right],$$ (3)

where ^^^ indicates the unit vector $\hat{a}=\frac{a}{|a{|}_{2}}$.

The maximum likelihood estimator (MLE) is used to evaluate a power-law relationship^28^

$$P\left(x\right)\propto x^{-\alpha },$$ (4)

where *P*(*x*) is the probability distribution function (PDF) for *n* strain relaxation events of size *x*, where *x* is taken as Δ*g_L_* or Δ*g_A_*. The scaling exponent *α* is computed as

$$\alpha =1+n{\left[\sum_{i=1}^{n}\;\text{ln}\;\frac{x_{i}}{x_{\text{min}}}\right]}^{-1}.$$ (5)

Evaluation of the MLE is carried out using the powerlaw package^29^ and listed in Table III.

In addition to the power-law, a power-law with an exponential cut-off (called truncated power-law here, *x*^−*α*^*e*^−*λx*^) and exponential (*e*^−*λx*^) distributions were fit. The log likelihood ratios, as described by Ref. 28 and implemented in Ref. 29, are also calculated. Positive values of the likelihood ratio indicate preference of the power-law fit in contrast to the alternative. A *p*-value is provided along with the corresponding likelihood ratio.

## III. RESULTS

### A. Intermittent plasticity in diffraction spot evolution

For both Ti-7Al and AZ31, it was possible to connect diffraction spots showing isolated transients to grains indexed by ff-HEDM.

#### 1. Ti-7Al

**FIG. 3.**
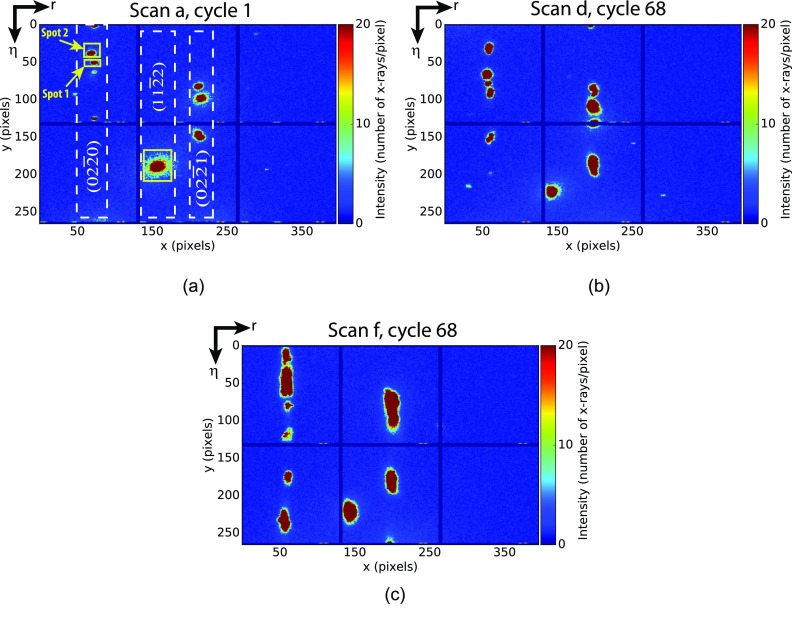
Evolution of diffraction spots captured on the MM-PAD, from (a) scan a, *ω* oscillation cycle 1 to (b) scan d, *ω* oscillation cycle 68 and (c) scan f, *ω* oscillation cycle 68. Note the (*hkil*) of the diffracting planes in (a). In (a), the spots surrounded with the yellow boxes are used for further analysis in this work.

Figure 3 shows the gradual change in shapes and sizes of diffraction spots over scan steps a, d, and f [as marked on Fig. 1(b)] in the radial-azimuthal (*r* − *η*) plane. These plots are generated using a maximum over all the 146 image frames collected using the MM-PAD during any particular *ω* oscillation cycle [cycle numbers are noted in Figs. 3(a)–3(c)]. Diffraction spots captured on the MM-PAD correspond to {02.0}, {11.2}, and {02.1} families of lattice planes, as indicated in Fig. 3(a). Generally speaking, diffraction spots tracked using the MM-PAD showed different character in the evolution of spread in intensity likely due to variation in active dislocation types/slip systems in the different grains.^30,31^ For example, the spot labelled “Spot 1” underwent greater spread in the *η* direction as compared to “Spot 2” over the course of several stress relaxation steps.

**FIG. 4.**
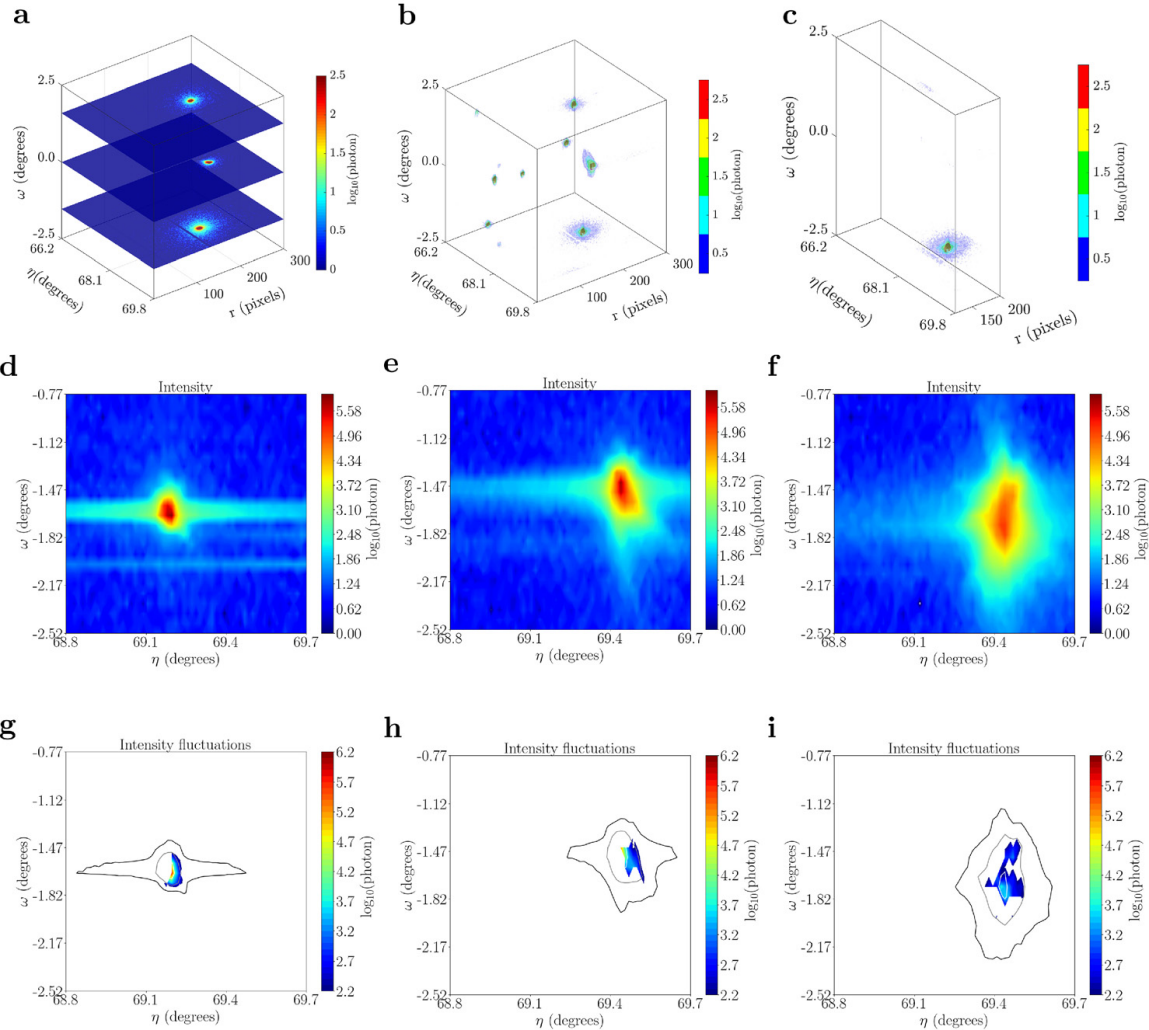
Evolution of diffraction spots captured on the MM-PAD shown in three dimensional r-*η*-*ω* space and two dimensional projections on the *η*-*ω* plane. (a) MM-PAD images stacked in *ω* for the stress relaxation step a. (b) The stack of images are merged together to describe diffractions spots in three-dimension. (c) Diffraction spot on the ring is selected for two-dimensional projection and analysis of intermittent plasticity. (d)–(f) Two dimensional projections on the *η*-*ω* plane for diffraction spot at stress relaxation steps a, d, and e. (g)–(i) Representative intensity fluctuations in the diffraction spot for stress relaxation steps a, d, and e.

Plastic deformation events are correlated with intensity fluctuations, at each point in a region of interest (ROI) around a particular diffraction spot on the *η*–*ω* plane. This particular rendering offers insight into the evolution of lattice orientation as a consequence of plastic slip. An example of the procedure to develop the *η*–*ω* projection is shown in Fig. 4. The diffraction spot from the {11.2} lattice planes was used for illustration in Fig. 4. Intensity changes between *η*–*ω* projections for consecutive loading steps concentrate mainly around the center of the diffraction spot, as given in Figs. 4(g)–4(i). Such changes in intensity were readily observable for the Ti-7Al sample.

With an indication of intermittency in hand, a next step is to relate spots on the MM-PAD to the stress state of the corresponding diffracting grain, and ultimately make a connection with the mode of crystallographic slip. Such a link was achieved for “Spot 1” of the {02.0} lattice plane family and the $\left(11\bar{2}2\right)$ spot, as indicated in Fig. 3.

**FIG. 5.**
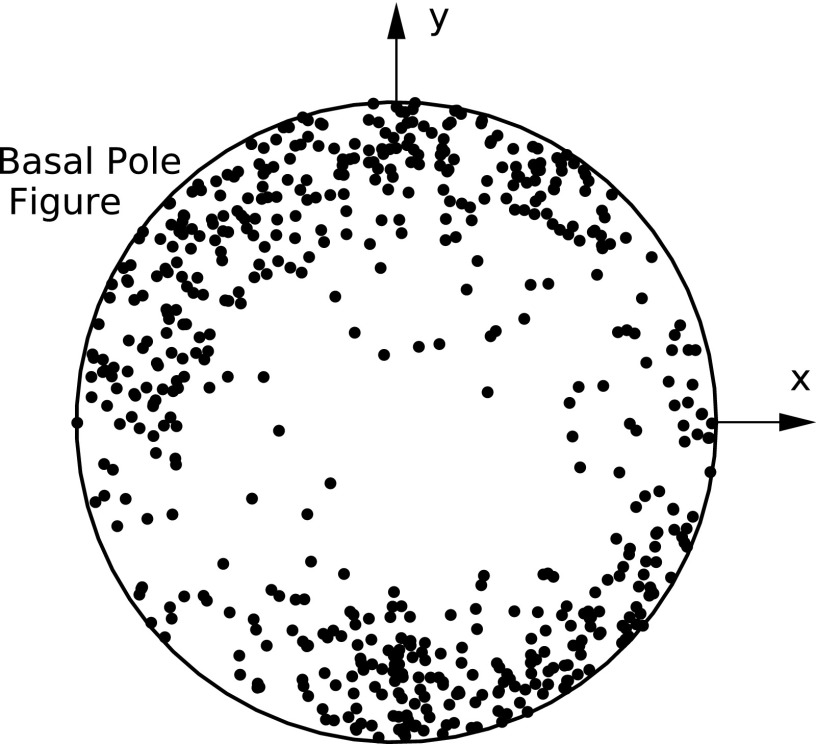
Basal pole figure for grains found from the analysis of ff-HEDM data for the Ti-7Al specimen.

**FIG. 6.**
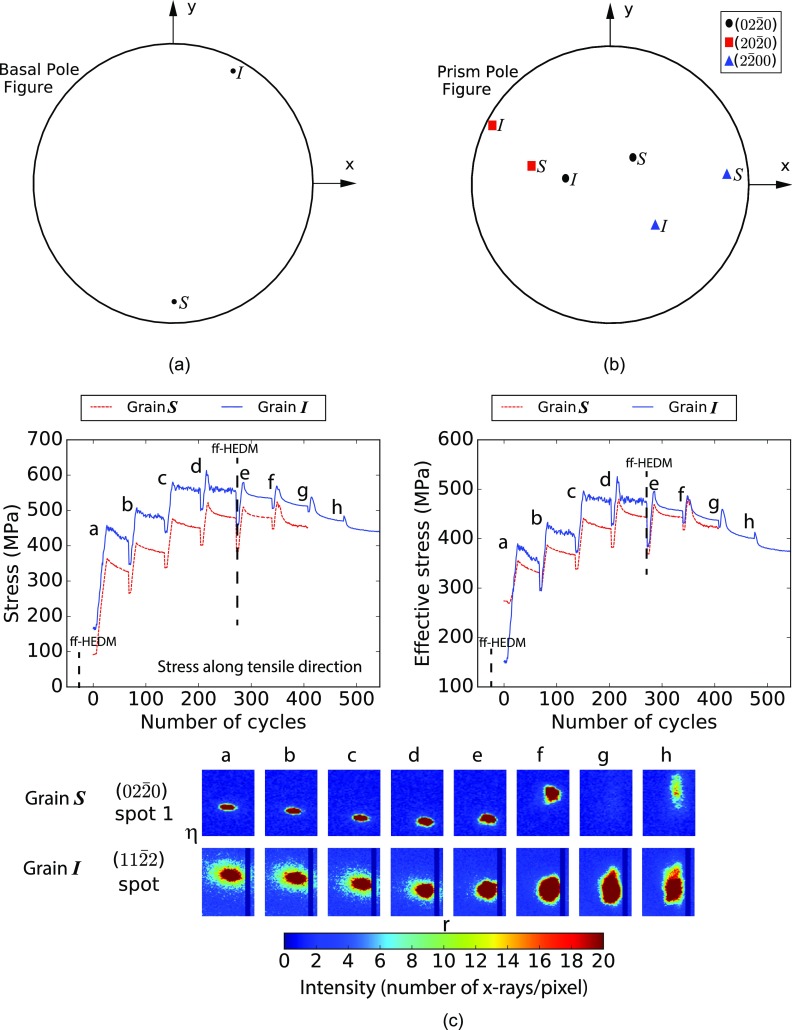
Transient response of the grains “*S*” and “*I*” during stress relaxation of Ti-7Al. (a) Basal pole figure for the two grains. (b) Prism pole figure for the two grains. (c) Stress relaxation response developed by following the diffraction spot center-of-mass the evolution of spots in the *η* − *r* plane. By scaling the stress tensor, developed through ff-HEDM, stress in evaluated along the tensile direction.

**FIG. 7.**
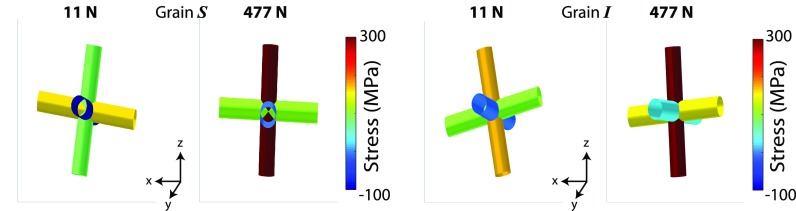
Stress jacks at loads ∼11 N and ∼477 N for grains (a) “*S*” and (b) “*I*” in the Ti-7Al specimen.

Scans for ff-HEDM, collected at load steps with an applied force of ∼11 N and ∼477 N [as marked in Fig. 1(b)], were analyzed to fit grain orientations and strains. The initial grain orientations were determined from the ff-HEDM scan performed at lower load [see Fig. 1(b)], and the same grains were followed in the deformed state to find their strains using hexRD, stresses were determined thereafter. Figure 5 shows the basal pole figure for all the grains found at lower load; the majority of the grains have non-basal orientations. Grains “*S*” and “*I*,” associated with $\left(02\bar{2}0\right)$ (Spot 1) and $\left(11\bar{2}2\right)$ spots, respectively, were found with reasonable completeness and fitting error (Table I). Both of these grains have the $\left(02\bar{2}0\right)$ prismatic plane normals only slightly tilted with respect to the tensile direction (z-direction) and the basal axis along sample y [Figs. 6(a) and 6(b)]. The stress jacks given in Figs. 7(a) and 7(b) provide insight into the multiaxial stress state of the grains. Under loading of the sample to 477 N, grain “*S*” aligns a bit more closely with applied macroscopic stress, as compared to grain *“I*.”

**TABLE I.** Basal and prismatic resolved shear stresses (RSS) (absolute values) for grains “*S*” and “*I.*”

Grain label	“*S*”		“*I*”	
Slip system	11 N	477 N	11 N	477 N
**Prismatic** (MPa)				
$\left(10\bar{1}0)[\bar{1}2\bar{1}0\right]$	29	87	25	**172**
$\left(0\bar{1}10)[2\bar{1}\bar{1}0\right]$	12	95	36	**167**
$\left(\bar{1}100)[\bar{1}\bar{1}20\right]$	41	**182**	10	5
**Basal** (MPa)				
$\left(0001)[2\bar{1}\bar{1}0\right]$	32	19	22	38
$\left(0001)[\bar{1}2\bar{1}0\right]$	57	78	11	29
$\left(0001)[\bar{1}\bar{1}20\right]$	89	59	12	67
*χ*^2^	0.0035	0.0052	0.0091	0.0072
Completeness	0.574	0.527	0.793	0.670

The stress relaxation behavior of the two grains was developed by interpolating between the stress tensor measured with ff-HEDM before and after the stress relaxation using the radial displacements (lattice strains) of the corresponding (MM-PAD) diffraction spots. For a cycle *i*, the stress tensors for a particular grain $\sigma^{i}$ during stress relaxation between $\sigma^{ini}$ (∼11 N) and $\sigma^{fin}$ (∼477 N) are scaled by the current radial position of the MM-PAD spot center-of-mass, *r^i^* as

$$\sigma^{i}=(\sigma^{fin}-\sigma^{ini})\frac{r^{i}-r^{ini}}{r^{fin}-r^{ini}}+\sigma^{ini},$$ (6)

where *r^ini^* and *r^fin^* are the MM-PAD spot center-of-mass positions at the time of the ff-HEDM measurements.

The stress relaxation behavior and spot spreading in the *η*-r plane indicate plastic activity in grains “*S*” and “*I*” (Fig. 6), with spreading particularly evident from relaxation step d forward. This may also be seen from the evolution of the $\left(11\bar{2}2\right)$ spot in the *η*–*ω* plane (Fig. 4). Small oscillations in the stress—associated with motion of the spot center-of-mass—accompanied with significant diffuse scattering in the diffraction spot are present in scan steps a-d for grain “*I*” [Fig. 6(c)]. Note that the diffuse scattering at the edges of the diffraction spots is reduced with increasing load. This may be associated with the destruction of previously observed short-range order^32^ with increasing plastic deformation. In contrast, grain “*S*” shows a smooth stress relaxation behavior; at the point of maximum loading, step d, there is comparatively less stress relaxation and spot broadening for grain “*S*” relative to grain “*I*” [Fig. 6(c)]. However, in step f, the deformation accelerates significantly in grain “*S*” and the spot moves out of the MM-PAD image frame—likely a consequence of yield. Again, comparing the relative degree of stress relaxation, it is only in this load step f that grain “*S*” shows a stress decrease larger that grain “*I*.” An interesting point to note is that with greater plastic deformation, both the spots translate along *η*, further indication of macroscale deformation of the sample and grain rotation in these latter loading steps.

The resolved shear stresses for the grains are provided in Table I—these values indicate that the grains mainly deform via prismatic slip. At the higher load, two different prismatic slip planes of grain “*I*” appear to develop relatively high resolved shear stresses (172 MPa and 167 MPa), nearing the critical resolved shear stress. On the other hand, for grain “*S*,” such high resolved shear stresses are found only in one of the prismatic slip planes (Table I). For comparison, the strength of the prism and basal slip systems in tensile loading are ∼248 MPa and ∼253 MPa, respectively.^33^ A lower value of strength would be expected in this relaxing state, as a consequence of rate sensitivity.

#### 2. AZ31

**FIG. 8.**
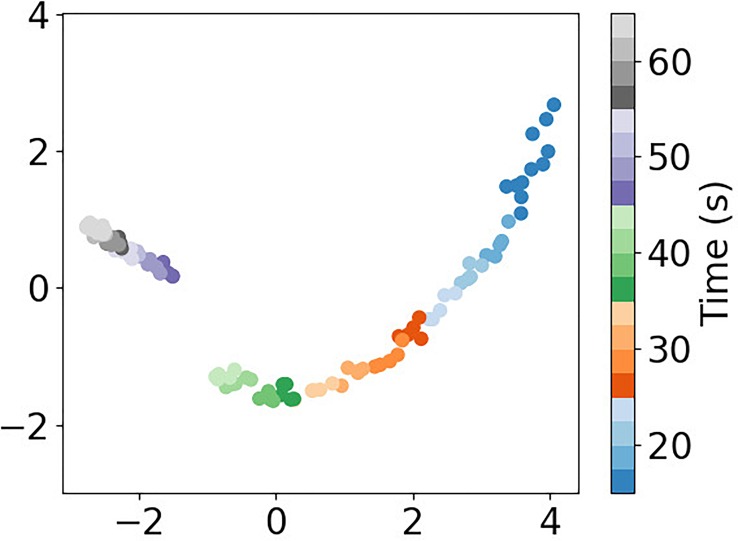
Dimension reduction of diffraction images measured during stress relaxation in the AZ31 specimen using the MM-PAD, showing change of manifold at ∼ 45 s. Axis units are arbitrary.

The sample AZ31 was loaded in small displacement increments until stress relaxation became evident from drops in load after a displacement increment. As compared to the Ti-7Al, the AZ31 material had many more, but less intense, diffraction spots—indicative of a finer grain size. Cursory examination of diffraction images provided scant evidence of isolated transients. However, insight into the presence of intermittency was found through the technique of dimension reduction. Individual detector images were reduced in size by removing pixels between reflections. This resulted in a total of 1,638,120 pixels for each oscillation of period 0.5 s. The 1.6M element “vectors” for each cycle served as input to a Principal Components Analysis (PCA).^27^ In particular, the relaxation shown in loading step d of Fig. 1(c) rendered an interesting result, given in Fig. 8. There is evidence of a transient about 45 s into the load and relaxation sequence.

**FIG. 9.**
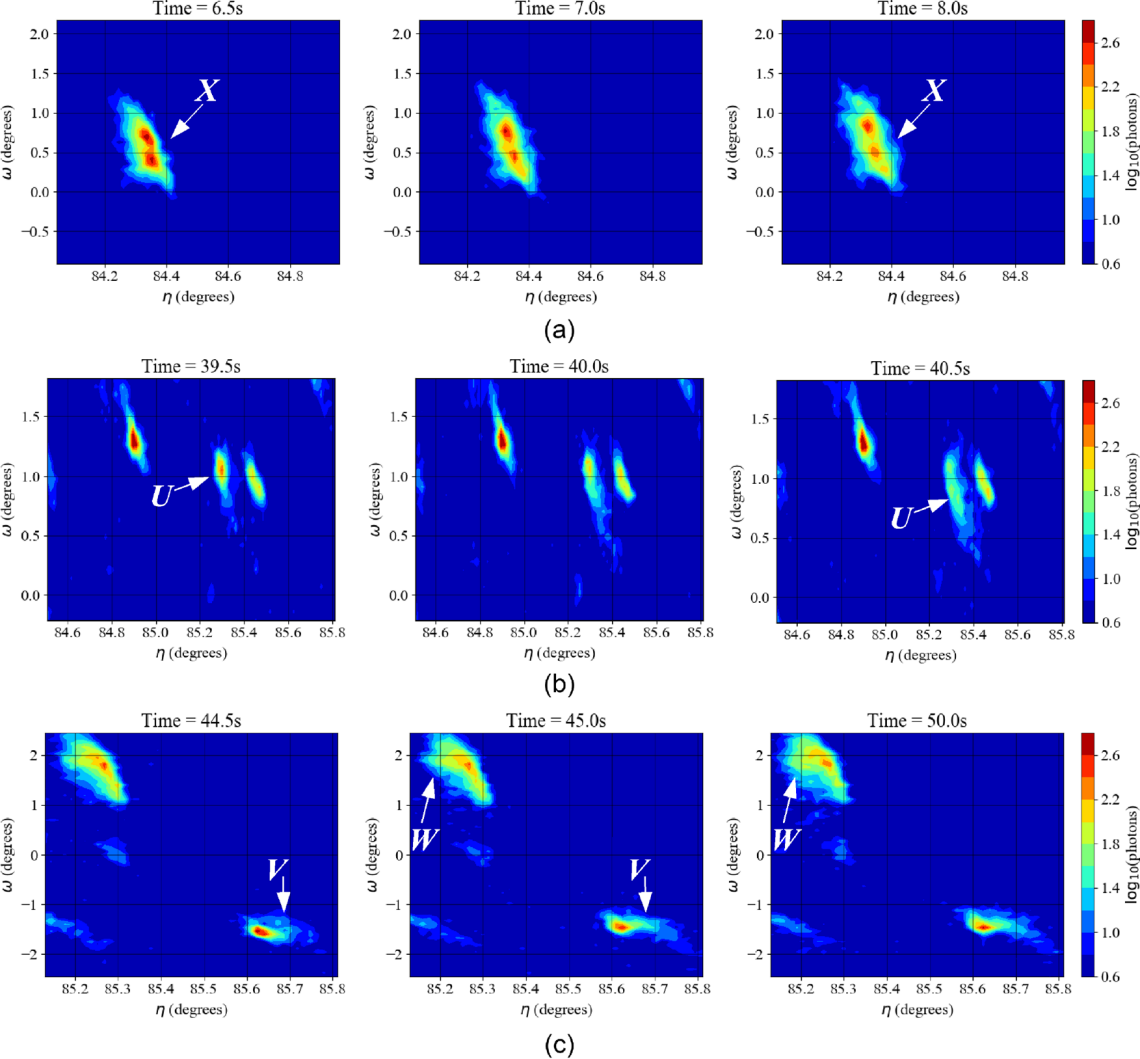
Evolution of diffraction spots showing distinct transient events of varying magnitude: (a) Further splitting of a spot “*X*” from the {00.4} lattice planes upon loading (b) Spreading of a spot labelled “*U*” from the {02.1} lattice planes at ∼40 s (c) Relatively slight spreading of two diffraction spots “*V*” and “*W*” from the {11.2} lattice planes (note these are from different grains) at roughly 45 and 50 s, respectively. Multimedia view: https://doi.org/10.1063/1.5068756.1
10.1063/1.5068756.1

With guidance in hand, instances of transient events were found in data for step d, as shown in Fig. 9. There are a sequence of transient events, occurring around the time indicated by the PCA analysis. Looking at Fig. 9(b) (Multimedia view), there is a spot, labelled “*U*,” that spreads primarily in *ω* at ∼ 40 s, with concomitant loss of intensity. Despite the spread of the diffraction spot, the *integrated* intensity of this spot actually remained constant, with an average of 2345 ± 68 photons before and 2313 ± 55 photons following the transient. At 45 s, there is a shift in *ω* and a relatively small degree of spreading in *η* of spot “*V*,” shown in Fig. 9(c). Finally, spot “*W*” shows very slight spreading in *η* at 50 s.

Rather fortuitously, images for ff-HEDM were collected immediately following this particular step. Searching along fibers corresponding to spots labeled *U-X* in Fig. 9, corresponding grains were indexed and the elastic strain tensors were determined, from which stresses were calculated. The maximum resolved shear stresses on the basal and 2nd order pyramidal slip systems are given in Table II. During application of the load, there was a splitting of a {00.4} spot, “*X”* of Fig. 9(a). The resolved stress for basal slip for this grain is larger than the average value (Table II). Of particular significance, the spot showing the most distinct intermittency in relaxation, “*U*,” is associated with a large resolved shear stress of 181.8 MPa for the 2nd order pyramidal slip.

**TABLE II.** Resolved shear stress for AZ31 from ff-HEDM analysis.

Grain ID	Reflection	Basal (MPa)	Pyr. (2nd) (MPa)	Completeness	*χ*^2^
*U*	{02.1}	57.5	181.8	0.48	0.0023
*V*	{11.2}	34.2	43.4	0.56	0.0022
*W*	{11.2}	32.2	41.2	0.59	0.0019
*X*	{00.4}	54.1	58.5	0.39	0.0021
*avg (for AZ31)*	…	*39.6*	*52.9*		

### B. Analysis of intermittency

**FIG. 10.**
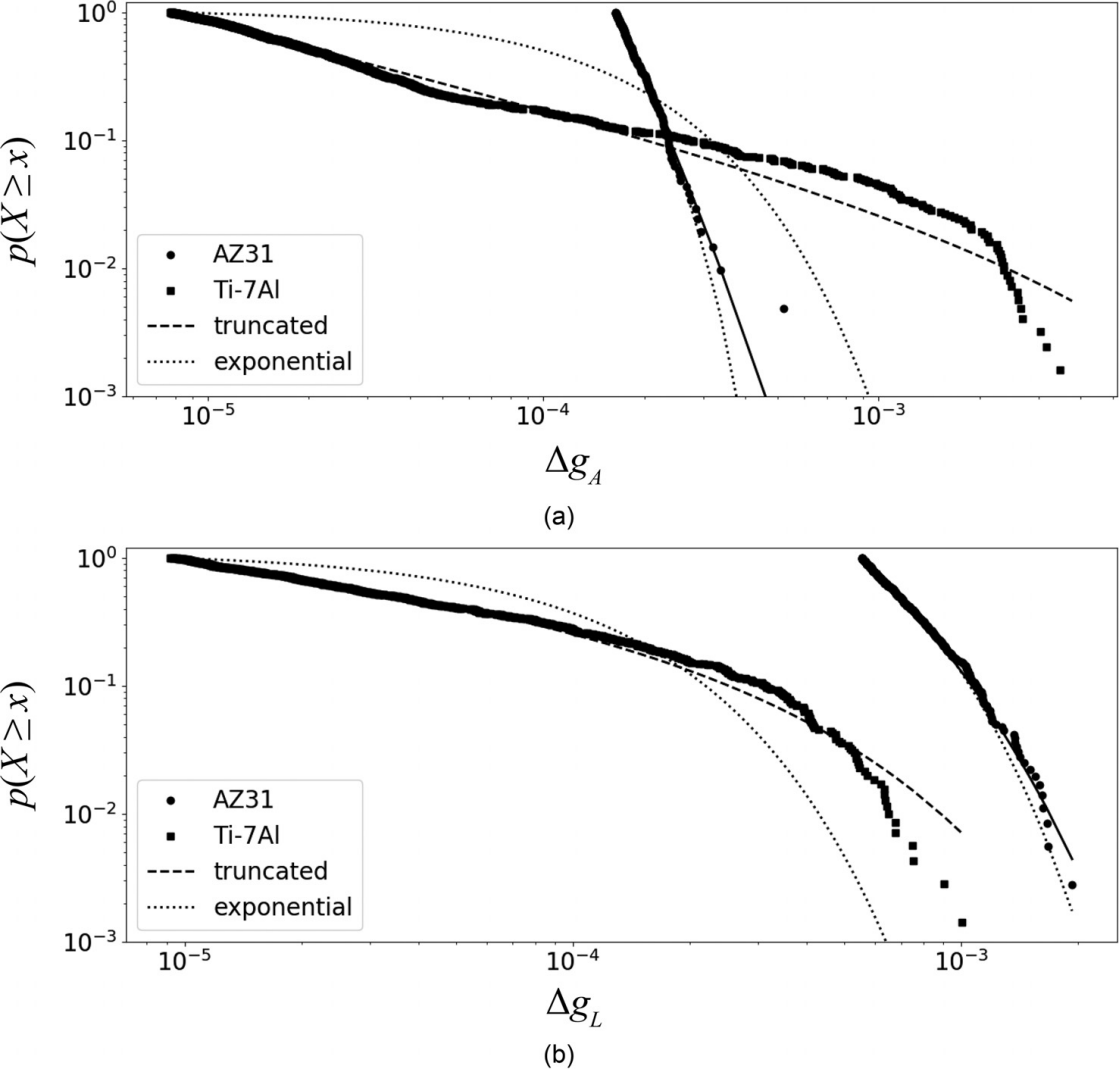
Complementary Cumulative Distribution Function of plasticity events corresponding to change in (a) diffraction vector angle and (b) diffraction vector length for the AZ31 and Ti-7Al specimens. The dashed and solid lines indicate fits using the truncated power law and exponential fits, respectively.

With the goal of characterizing intermittency, events from all diffraction spots were drawn from scans a, b, c, d, and e for the Ti-7Al sample [Fig. 1(b)] and scans d, e, f, and g for the AZ31 sample [Fig. 1(c)]. Using the procedure outlined above, only images taken during the dwell period were considered. The complementary cumulative distribution function (CCDF) for Δ*g_A_* and Δ*g_L_* are shown in Fig. 10. Scaling exponents are given in Table III, with exponents for a power law and truncated power law are given as *α* and *α_t_*, respectively.

**TABLE III.** Scaling exponents.

Material	*x*	*α*	*α_t_*	$\frac{1}{\lambda }$	Events	*R*_exp_ (p-value)	*R*_tpl_ (p-value)
AZ31	Δ*g_A_*	7.11 ± 0.43	5.89	1.9 × 10^−4^	206	1.61 (0.65)	−0.12 (0.63)
	Δ*g_L_*	4.41 ± 0.18	2.10	4.2 × 10^−4^	358	−1.97 (0.60)	−3.91 (0.005)
Ti-7Al	Δ*g_A_*	1.71 ± 0.020	1.66	1.0 × 10^−2^	1237	1352. (0.00)	−6.64 (0.00)
	Δ*g_L_*	1.62 ± 0.023	1.23	5.1 × 10^−4^	701	149. (0.00)	−52.8 (0.00)

For Ti-7Al, the exponent *α_t_* is similar for the diffraction vector angle and length change: 1.71 and 1.62 for Δ*g_A_* and Δ*g_L_*, respectively. In both cases, a truncated power law is supported, as *R*_tpl_ < 0 with *p *<* *0.01. Additionally, a power law is favored over an exponential distribution, *R*_exp_ > 0, again with *p *<* *0.01. The domain of scaling for Δ*g_A_* is larger than that found for Δ*g_L_*, offering indication that the angle change associated with the lattice plane rotation provides a preferable measure for characterizing plastic events. Further, the exponent for a truncated power law, *α_t_* = 1.66 is consistent with *α* = 1.71 for Δ*g_A_*. An indication of the cut-off of largest events is given by the parameter $\frac{1}{\lambda }$, adapted from the truncated power law *x*^−*α*^*e*^−*λx*^. The relatively large value of $\frac{1}{\lambda }$ for Δ*g_A_* reinforces the case for a truncated power law to characterize transients in plasticity of the Ti-7Al sample by angle changes in diffraction.

In the case of AZ31, the exponents are greater than those found for Ti-7Al with relatively fewer events contributing to the analysis. No argument can be made for a power law over an exponential fit for AZ31, as *p *>* *0.05 for *R*_exp_ in the case of both angle and length changes. Power law exponents are large relative to those found for Ti-7Al, with the exception of a truncated power law with *α_t_* = 2.1, with a negative likelihood ratio having *p *<* *0.01. The truncated power law parameter $\frac{1}{\lambda }$ is small for both length and angle changes.

The detail above is made clear by plots of the exponential distribution given in Fig. 10. An exponential function describes well the statistics for AZ31. In contrast, a truncated power law provides a much better fit for Δ*g_A_* statistics of the Ti-7Al material. In Fig. 10(a), an inflection in the data is observable at Δ*g_A_* = 0.00033 for Ti-7Al. Evaluating the MLE with *x*_min_ = Δ*g_A_* = 0.00033 gives an exponent of *α* = 1.66 ± 0.033, quite close to the value of 1.71 given in Table III. Such consistency in the exponent over a range of choice in minimum event size in computing the MLE offers further evidence for a (truncated) power law characterization of Δ*g_A_* for Ti-7Al.

We close this section with some words of caution. The scaling exponent, as well as character of the cut-off is affected by parameter choices taken in clustering. As an example, increasing the minimum cluster size leads toward truncation at a lower value. Said another way, averaging over larger regions in reciprocal space tends to smooth out events. Similar effects follow from the choice of threshold for decrease in photons between images. Other descriptions for truncation of the power law are possible.^34^ Finally, there is the difference in the general character of diffraction for the two alloys, intensities were much stronger for the (larger grain size) Ti-7Al. For these reasons, the authors caution against drawing any firm conclusion regarding the particular values of scaling exponents in Table III. Rather, we make the following inference: the results for Ti-7Al over a range of threshold and cluster parameter choices taken in the course of the data analysis, consistently showed evidence of a heavy-tailed distribution—particularly evident for the measure Δ*g_A_*. For the AZ31, again considering fits over a range of cluster parameter choices, there is no evidence that statistics follow a heavy-tailed distribution.

## IV. DISCUSSION

Evidence of intermittent plasticity during stress relaxation was found for both Ti-7Al and AZ31 through direct observation of intensity fluctuations, such as spreading and shifts of spot center of masses. Direct observations of intermittent dislocation motion was found from the evolution of distributions of diffracted intensity at distinct points during the stress relaxation process, while grain level stress relaxation were studied through a power law analysis of radial and azimuthal shifts within diffraction spots.

Fluctuations in intensity were readily apparent in detector image sequences for Ti-7Al. Two diffraction spots were tracked using the MM-PAD, one showing marked intermittency and another relatively quiescent during the initial relaxation steps, were related to average grain lattice strain through ff-HEDM. The diffraction spot showing intermittency was associated with a grain oriented for the slip on two prismatic systems. A more general statistical analysis, considering all diffracted intensity on the MM-PAD, offers support for the Ti-7Al having a power law scaling with exponential cut-off. A similar scaling response was found for this material in creep deformation, albeit at much lower rates of data collection.^3^ There is one point of note, however: the very large, collective bursts in the creep experiment occurred predominantly in grains having a high resolved shear stress for basal slip, though some isolated bursts were found in grains favoring prism slip.

For the AZ31 sample, intermittent events were not identified through a manual review of detector images, but only through visualization techniques of dimension reduction. Vectors representing stacks of images collected during relaxation were visualized in two dimensions through PCA. This made it possible to identify transient lattice rotation. In particular, three events—closely spaced, but distinct in time—were observed at the point in relaxation indicated by PCA. In agreement with the relative sparsity and evidence for power law scaling, even with a cut-off, the degree of intermittency is weaker for the AZ31.

Returning to Fig. 10(b), where the events are based on changes in the diffraction vector length, it is worth considering that despite the difference in the shapes of the distributions, the AZ31 events appear to be biased to larger magnitudes than that of the Ti-7Al. On the surface, this appears to be inconsistent with a lesser degree of intermittency for the AZ31, however, note both that an increase in the diffraction vector length corresponds to a decrease in the elastic strain and that Mg is a much more elastically compliant material than Ti;^25^ thus, for the same magnitude of stress drop, one would expect the more compliant material (AZ31) to undergo a larger decrease in elastic strain and correspondingly larger increase in the diffraction vector length. Further, the detector was positioned at a smaller *η* angle for the AZ31 sample [Fig. 1(a)]: a larger strain transient is expected as the diffraction vector becomes more aligned with the loading axis. By contrast, a change in the diffraction vector angle [Fig. 10(a)] is sensitive to lattice rotations and thus may be considered to be a more direct measure for plastic activity and in this case we see that indeed the distribution of Ti-7Al events includes events that are approximately an order of magnitude larger than that seen in the AZ31.

Our results may be viewed in light of the “mild to wild” proposal by Weiss *et al.*^35^ The preferred modes of basal and prism slip for the AZ31 and Ti-7Al alloys, respectively, do not admit a component of plastic deformation along the c-axis. However, the presence of pyramidal slip in stress relaxation at room temperature is indicated in the studies of AZ31.^19,36^ In the present work, one of the most distinctive relaxation events observed in the AZ31 sample was associated with a high resolved shear stress on the 2nd order pyramidal plane. While collective dislocation motion is likely present,^37^ the presence of a pyramidal slip mode would aid considerably in local relaxation at the grain scale. The availability of a pyramidal slip mode would act to shield long-range elastic interactions and interaction between dislocations. The relatively fine grain size of the AZ31 would further contribute to localizing relaxation and promoting a cut-off of the largest event size. There is no evidence for power law behavior in the AZ31 material; plastic events are “mild” fluctuations. In contrast, the tendency to planar slip is well-documented in the Ti-7Al used in this study, as a consequence of short-range order,^38,39^ and evidence of scale-free flow in the Ti-7Al (with a cut-off) is found in the present study. The conclusion is that “wild” fluctuations were found in Ti-7Al.

A direction for future research may be found in the diffuse scattering signal for the $\left(11\bar{2}2\right)$ diffraction spot associated with grain “*I*,” as shown in Fig. 6. As noted above, this diffuse scattering character is somehow associated with the presence of small oscillations in the stress plot, due to back-in-forth shifts of the spot center-of-mass in 2*θ*. This diffuse character decreases after relaxation step e—upon yield, as indicated by the spread of spots in Fig. 4, scan f—along with a smooth character in the stress response. As noted above, effects such as the weak pairing of dislocations associated with the presence of the short-range order^32^ may play a role here. Looking forward, high energy x-ray diffraction studies provide a pathway beyond identifying intermittency and relating to driving forces, to understanding of the evolution of the dynamics of the dislocation structure in plastic deformation.

## V. SUMMARY

• The characterization of intermittency within individual deforming grains is achieved combining macroscopic stress relaxation data, the resolved shear stress found using ff-HEDM, and rapid x-ray diffraction spot measurements made using a Mixed-Mode Pixel Array Detector (MM-PAD).
• The evolution of the dislocation content within the deforming grains is monitored following intensity distributions in reciprocal space.
• Intermittent bursts of plasticity are observed in both Ti-7Al and the magnesium alloy AZ31.
• Results for the Ti-7Al alloy show the presence of large stress fluctuations in contrast to AZ31, which shows a lesser degree of intermittent plastic flow.

## Figures and Tables

**FIG. 11.**
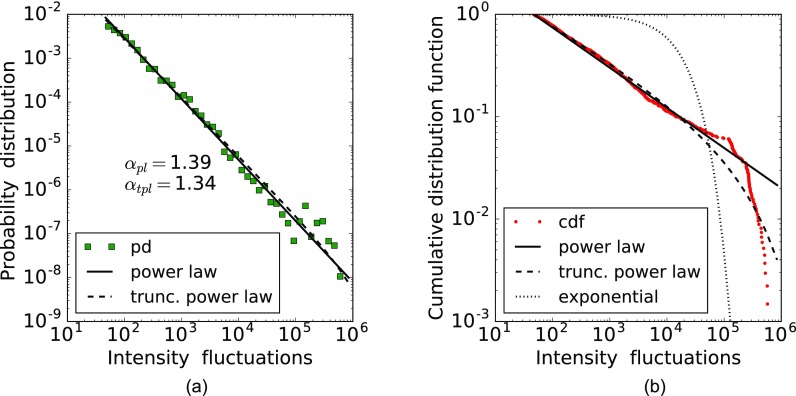
Scaling behavior of probability distribution vs. magnitudes of intensity fluctuations in a ROI around the $\left(11\bar{2}2\right)$ spot. (a) The Probability Distribution Function (PDF). (b) The Complementary Cumulative Distribution Function (CCDF).

## APPENDIX: SCALING OF INTENSITY FLUCTUATIONS

The analysis procedure taken in Sec. III, above, does not consider the effect of intensity, beyond weighting in computing the diffraction vector, lending the capability to develop statistics across different (hk.l) reflections and different grains. As a point of comparison, it is interesting to examine statistics of fluctuations for a single diffraction spot from a plastically deforming grain: the grain “*I*” associated with the $\left(11\bar{2}2\right)$ spot, as highlighted for the Ti-7Al material.

The diffraction rings are projected on the *η*-*ω* plane in a similar way as was done for the g-vector approach. Two or more consecutive intensity fluctuations (at a point in the ROI) are above a threshold of 20 x-rays are required for an event. Those intensity fluctuations are summed to give the magnitude of the plasticity event. Any intensity fluctuations below 20 x-rays are discarded. The regime of scaling, shown in Fig. 11, extends over three orders of magnitude. There are a total of 1351 fluctuation events that contribute to the scaling. A truncated power law is supported with $R=\text{ln}\;(L_{pl})-\text{ln}\;(L_{\;\text{exp}\;})=3376$ and *p *=* *0.00.
